# Trousseau syndrome with intrahepatic cholangiocarcinoma that could be removed radically after endovascular treatment: Report of a case

**DOI:** 10.1002/brb3.1660

**Published:** 2020-06-07

**Authors:** Shuei Murahashi, Yosuke Takeuchi, Shintaro Hayashida, Yuki Ohya, Seigo Shindo, Tadashi Terasaki, Yukihiro Inomata, Yasuyuki Hara

**Affiliations:** ^1^ Department of Neurology Kumamoto Rosai Hospital Kumamoto Japan; ^2^ Department of Pediatric Surgery Kumamoto Rosai Hospital Kumamoto Japan; ^3^ Department of Neurology Kumamoto Sekijyuji Hospital Kumamoto Japan

**Keywords:** endovascular treatment, intrahepatic cholangiocarcinoma, stroke, trousseau syndrome

## Abstract

**Background:**

Trousseau syndrome is a poor prognosis. We report a case of Trousseau syndrome treated by radical resection after endovascular treatment.

**Case:**

A 59‐year‐old woman presented to our department reporting spontaneous dizziness and pain of the upper abdomen. Magnetic resolution imaging (MRI) showed shower embolization of Brain. Contrast‐enhanced computer tomography (CT) showed renal infarction and splenic infarction, and a tumor was observed in the retrohepatic area. On day 9, sudden right side joint prejudice, neglect of left half space, and left hemiplegia were observed. MRI revealed obstruction of the right middle cerebral artery (MCA) perfusion zone. On the same day, endovascular treatment was performed and reperfusion was obtained. We decided on a radical surgery policy because there were a primary lesion and a high risk of new embolism, and no metastasis was seen.

**Discussion:**

Trousseau syndrome generally has a poor prognosis, but active treatment should be considered as an option when we can expect the recovery of function.

## Graphical abstract

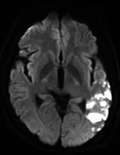

## 1 INTRODUCTION

Trousseau syndrome is known as embolism associated with a malignant tumor. And its prognosis is known as poor (Chen, Lin, Lin, Chen, & Cheng, 2011). Thromboembolism in the lower extremities, lung, brain, heart, kidneys, or spleen may lead to death among patients with this syndrome (Nakayama, Iha, & Kanazawa, 2002). The syndrome can be treated by curative tumor resection with curative therapy, but when patients present with advanced cancer and curative therapy cannot be used (Nakayama et al., 2002). We have experienced a case that Trousseau syndrome with intrahepatic cholangiocarcinoma that could be removed radically after endovascular treatment.

## 2 CASE REPORT

**Figure 1:**
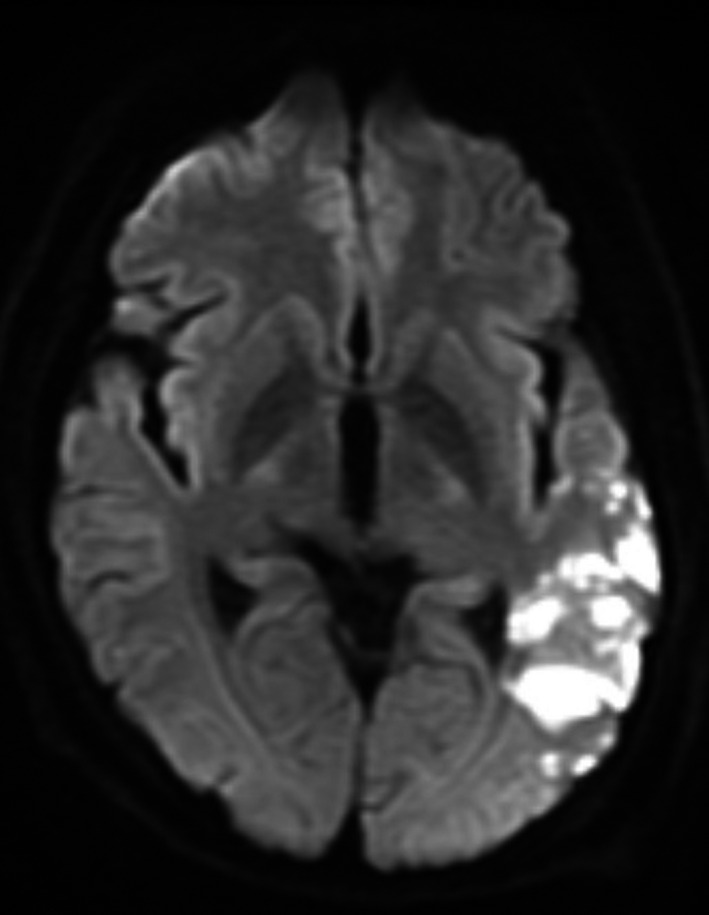
Image diagnosis of the case. Brain MRI showed shower embolization under the cortex of the left temporal lobe and parietal lobe

**Figure 2:**
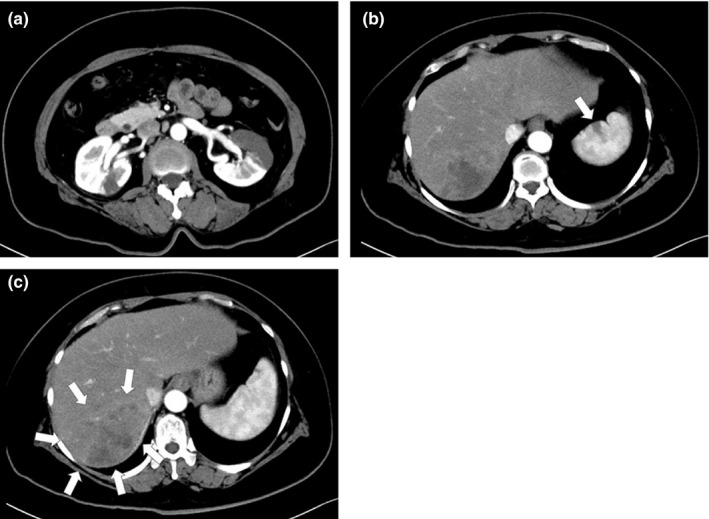
Image diagnosis of the case. (a) Contrast‐enhanced CT showed evidence of bilateral renal infarction. (b) Contrast‐enhanced CT showed splenic infarction (pointed by arrow head). (c) Contrast‐enhanced CT showed a tumor in the retrohepatic area(pointed by arrows heads)

**Figure 3:**
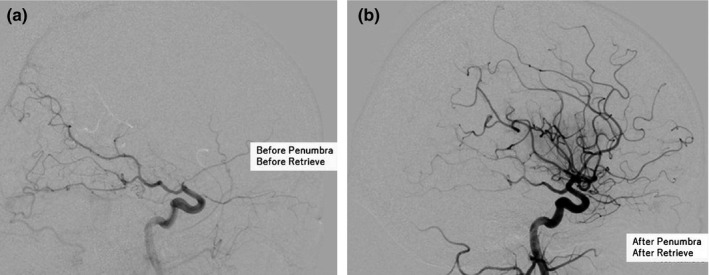
Images of vessel occlusion. (a) Obstruction was confirmed at the proximal part of the right middle cranial artery. (b) After thrombectomy, complete reperfusion was obtained

A 59‐year‐old woman saw her family doctor with upper abdominal pain, nausea, and dizziness and presented to our hospital. She had never pointed out of atrium fibrillation or other heart diseases. From neurological examination, no significant neurological symptom was observed except mild dysmetria and decomposition, but those findings were equivocal. Brain Magnetic resolution imaging (MRI) revealed shower embolization under the cortex of the left temporal lobe and parietal lobe (Figure 1). Contrast‐enhanced whole‐body computer tomography (CT) showed renal infarction and splenic infarction (Figure 2a,b), and a tumor was observed in the retrohepatic area (Figure 2c). From Laboratory data, d‐dimer was elevated (46.3 ng/ml) and tumor maker (CA 19–9) was extremely elevated (>120,000 U/dl). Other tumor maker was negative. Because Trousseau syndrome was strongly suspected, heparin 10,000 U/day administration was started after hospitalization. The tumor was suspected of intrahepatic cholangiocarcinoma. On day 9, sudden right side joint prejudice, neglect of left half space, and left hemiplegia were observed. National Institutes of Health Stroke Scale (NIHSS) score was 18. MRI revealed ischemic stroke; obstruction of proximal of the right middle cerebral artery (MCA). On the same day, endovascular treatment was performed and reperfusion was obtained (Figure 3).

Thrombolysis in Cerebral Infarction (TICI) was Grade 3.

### 2.1 Vessel occlusion findings

Sheath: 9Fr(femoral).

Guiding catheter: 9Fr Branker®

Inner catheter: 8Fr JB2 countdown®

Micro catheter: Penumbra 5MAX ACE®, Velocity®

Micro guidewire: CHIKAI 14®

Clot retriever: Solitaire 6x40®

The right femoral artery was punctured, and the 9Fr long sheath was placed. Branker® was guided to the right internal carotid artery with 9Fr Branker®/6Fr JB2 countdown®/35 Angle® and photographed. Obstruction was confirmed at the proximal part of the right MCA. We tried lesion cross, but it was difficult because thrombosis was very hard. Selective imaging was performed after leading the wire and guiding Velocity® to M2. We expand Solitaire 6x40 and angiography was performed to confirm the position of the thrombus. We guided Penumbra 5MAX ACE68® and started suction. After 90 s, clot retrieve succeeded. Thrombolysis in Cerebral Infarction(TICI) was Grade 3.

Onset to puncture: 12:30.

Onset to repurfusion: 13:42.

After endovascular treatment, joint prejudice and neglect of hemilateral space disappeared but hemiplegia remained. NIHSS score improved to 9. While joint prejudice and neglected spatial neglect improved, we decided on a radical surgery policy because there were a primary lesion and a high risk of new embolism, and no distant metastasis or invasion was seen on the image. Right hepatectomy and partial diaphragmatic resection were performed. The postoperative course was good. After the operation, Heparin 10,000 U/day administration had continued till D‐dimer inversion. The patient was transferred to rehabilitation without recurrence of cerebral infarction. The pathological examination showed the thrombosis was fibrin thrombosis. Tumor was intrahepatic cholangiocarcinoma: moderately differentiated adenocarcinoma.

## 3 DISCUSSION

Trousseau syndrome is a paraneoplastic syndrome that produces neurological symptoms associated with latent malignant tumors. This condition is recognized to cause systemic thrombosis as well as brain infarction due to enhancement of the coagulant system induced by the malignant tumor (Uchiyama, Terashi, Shimizu, Hashimoto, & Iwata, 2005). Mucin‐producing adenocarcinoma is a frequent histologic type in such cases (Sutherland, Weitz, & Liebman, 2003; Umehara, Nomoto, & Yanazume, 2006).

Trousseau syndrome associated with cholangiocarcinoma is rare. A total of 11 case reports of cholangiocarcinoma with Trousseau syndrome are published in English (Blum et al., 2016). Of the 12 cases, including this one, the most common presenting thromboembolism was deep vein thrombosis (4 cases) followed by pulmonary embolism (3 cases). Portal vein thrombosis was ultimately uncovered in 4 of the 12 cases. Splenic thrombosis, stroke, cardiac valve vegetations, and superficial thrombophlebitis were also reported in at least 3 cases. Only 4 patients, including this one, survived for more than 3 months following their initial presentation of thrombosis, which probably reflects both the poor overall prognosis for cholangiocarcinoma (5‐year survival, 5%–10%) and the advanced stage of disease when patients present with thrombosis (Anderson, Pinson, Berlin, & Chari, 2004).

Diagnosis of cholangiocarcinoma was made during autopsy for six cases and by CT or ultrasound‐guided liver biopsy, fine needle aspiration, or endoscopic retrograde cholangiopancreatography punch biopsy in the remaining cases. This case was the only one pathologically diagnosis after radical resection.

The first important point of this case is balancing the need for anticoagulation in managing multiple thrombosis and hypercoagulation with the need for a radical resection. The initial treatment modalities for cancer‐associated thrombosis include low molecular weight heparin (LMWH), unfractionated heparin (UFH), and fondaparinux. A meta‐analysis of randomized controlled trials found that, compared with UFH, LMWH was associated with a statistically significant reduction in mortality after 3 months of treatment [relative risk = 0.71; 95% confidence interval (CI) = 0.52–0.98] without an increased risk of bleeding (Akl et al., 2011). We chose UFH because of resectability of the tumor, but unfortunately recurrence of brain infarction occurred. This is probably because hypercoagulation was very accelerated considering of the level of CA19‐9 and D‐dimer.

The second important point of this case is endovascular treatment. In general, endovascular treatment is rare in ischemic brain infarctions associated with Trousseau syndrome (Sakuta, Mukai, Fujii, Makita, & Yaguchi, 2019). The reasons are activity of daily living(ADL), location of stroke, time from onset, and so on. In this case, it was considered to be an indication for endovascular treatment because it was an in‐hospital onset and obstruction of the main artery, and the patient had been independent of ADL.

There are seldom reports that refer to anticoagulation after radically resection of tumors associated with Trousseau syndrome. Moreover, there are no indicators when to stop anticoagulation therapy. D‐dimer is the result of dissolving of the thrombus. CA19‐9 does not directly indicate tumor hypercoagulation. Both were used as tentative indicators, but further research would be required when we stop anticoagulation after radically resection.

From this case, it has proven that some cases of Trousseau syndrome with intrahepatic carcinoma could be treated by radical operation and endovascular treatment though the prognosis of Trousseau syndrome is generally poor. The point we emphasize is the crucial point is ADL. In this case, predisease ADL was self‐sustaining(modified Rankin Scale 0), and prompt treatment for nosocomial onset cerebral infarction was likely to have a favorable neurological outcome. In general, endovascular treatment is indicated by premorbid ADL of modified Rankin Scale 0 or 1 (Powers, 2015). In this case, hemiplegia remained, but postoperative ADL independence was expected at the start of treatment. Anticoagulant therapy was also introduced from viewpoint of ADL after the operation. It was thought that lowering the possibility of reinfarction as much as possible could lead to ADL improvement.

The decision of endovascular treatment, operation, and anticoagulant therapy was not strictly based on evidences because there are few cases or studies. Further research is expected.

## CONFLICT OF INTEREST

All of authors have no conflicts of interest.

## AUTHOR CONTRIBUTIONS

Shuei Murahashi and Yosuke Takeuchi conceived of the presented idea. Seigo Shindo and Tadashi Terasaki performed endovascular treatment. Shintaro Hayashida, Yuki Ohya, and Yukihiro Inomata performed surgical operation. Yasuyuki Hara supervised the project.

## ETHICAL STATEMENT

The study was conducted with the consent of all patients following the Helsinki Declaration.

## Data Availability

The datasets generated and analyzed during the current study are available from the corresponding author on reasonable request.
